# The Impact of SST and PV Interneurons on Nonlinear Synaptic Integration in the Neocortex

**DOI:** 10.1523/ENEURO.0235-21.2021

**Published:** 2021-09-07

**Authors:** Christopher Dorsett, Benjamin D. Philpot, Spencer LaVere Smith, Ikuko T. Smith

**Affiliations:** 1 Neuroscience Center, Neurobiology Curriculum, University of North Carolina, Chapel Hill, North Carolina 27599; 2 Department of Cell Biology and Physiology, Carolina Institute for Developmental Disabilities, University of North Carolina, Chapel Hill, North Carolina 27599; 3 Department of Electrical & Computer Engineering, University of California, Santa Barbara, Santa Barbara, California 93106; 4 Neuroscience Research Institute, University of California, Santa Barbara, Santa Barbara, California 93106; 5 Department of Molecular, Cellular, and Developmental Biology, University of California, Santa Barbara, Santa Barbara, California 93106; 6 Department of Psychological & Brain Sciences, University of California, Santa Barbara, Santa Barbara, California 93106

**Keywords:** dendrites, inhibition, interneurons, nonlinearity, synaptic integration

## Abstract

Excitatory synaptic inputs arriving at the dendrites of a neuron can engage active mechanisms that nonlinearly amplify the depolarizing currents. This supralinear synaptic integration is subject to modulation by inhibition. However, the specific rules by which different subtypes of interneurons affect the modulation have remained largely elusive. To examine how inhibition influences active synaptic integration, we optogenetically manipulated the activity of the following two subtypes of interneurons: dendrite-targeting somatostatin-expressing (SST) interneurons; and perisomatic-targeting parvalbumin-expressing (PV) interneurons. In acute slices of mouse primary visual cortex, electrical stimulation evoked nonlinear synaptic integration that depended on NMDA receptors. Optogenetic activation of SST interneurons in conjunction with electrical stimulation resulted in predominantly divisive inhibitory gain control, reducing the magnitude of the supralinear response without affecting its threshold. PV interneuron activation, on the other hand, had a minimal effect on the supralinear response. Together, these results delineate the roles for SST and PV neurons in active synaptic integration. Differential effects of inhibition by SST and PV interneurons likely increase the computational capacity of the pyramidal neurons in modulating the nonlinear integration of synaptic output.

## Significance Statement

The principal function of neurons is to integrate a barrage of synaptic inputs and convert them into spike output. Such integration of inputs in the sensory neocortex ensures the transformation of environmental stimuli into a meaningful perception of the outside world. Synaptic integration in neuronal dendrites is shaped by passive electrical properties, active voltage-gated mechanisms, and inhibition from interneurons. Our results show that two genetically distinct subtypes of interneurons exert different types of inhibitory influence on active dendritic integration. Subtype-specific inhibitory influences provide a modulatory repertoire for the single-cell computations that occur during synaptic integration.

## Introduction

The integration of excitatory synaptic inputs in neuronal dendrites involves passive properties and voltage-gated active mechanisms (Spruston et al., 2016). Active mechanisms have been implicated as an important contributor toward diversifying postsynaptic responses in a number of behavioral contexts (Xu et al., 2012; Smith et al., 2013; Takahashi et al., 2016; Schmidt-Hieber et al., 2017; Sheffield et al., 2017), brain regions (Lavzin et al., 2012; Gale and Murphy, 2016; Sheffield et al., 2017), and animal species (Murayama and Larkum, 2009; Wilson et al., 2016). Furthermore, synaptic inhibition is a key component in sculpting and refining cortical activity (Silberberg and Markram, 2007; Fino and Yuste, 2011; Palmer et al., 2012; Ebina et al., 2014; Karnani et al., 2016) and behavior (Adesnik et al., 2012; Gentet et al., 2012; Kato et al., 2015; Sachidhanandam et al., 2016; Takahashi et al., 2016). Yet, it remains unclear how inhibitory interneurons modulate active dendritic processes during synaptic integration.

Inhibitory neurons are diverse in their morphology and connectivity, suggestive of their correspondingly diverse roles in neural circuitry. Interneurons exhibit a wide variety of axonal projection patterns onto their pyramidal cell targets. Basket cells are known to predominantly target cell bodies (Karube et al., 2004), while Martinotti cells target apical dendritic tufts (Wang et al., 2004). Layer 1 cells, including elongated neurogliaform cells, target superficial apical dendritic tufts (Jiang et al., 2013; Lee et al., 2015; Schuman et al., 2019), and chandelier cells target the axon initial segment (Kawaguchi and Kubota, 1997). Thus, inhibition can be either proximal or distal relative to the site of excitatory input, and this spatial relationship influences their functional interaction. Considering the passive cable property of the dendrites, proximal inhibition is effective at diminishing the amount of charge propagated to the soma (Koch et al., 1983; Vu and Krasne, 1992; Liu, 2004), whereas distal inhibition is less effective (Liu, 2004; Hao et al., 2009) and could be overcome by larger excitatory inputs (Vu and Krasne, 1992).

Inhibition can influence active dendritic synaptic integration as well (Palmer et al., 2012). Contrary to the passive case, a recent modeling study has demonstrated that the interaction between inhibition and active dendritic mechanisms is more effective for distal “off-path” inhibition than proximal “on-path” inhibition (Gidon and Segev, 2012). The NMDA receptor is a key component in active dendritic integration (Palmer et al., 2014; Stuart and Spruston, 2015). Computational modeling suggests that NMDA spikes are particularly sensitive to distal dendritic inhibition. When colocalized to the same dendritic segment, even small inhibitory conductances are capable of eliminating the nonlinear increase in membrane potential associated with NMDA spikes, while somatically placed inhibition had negligible effects on both the spike waveform at the dendrite and the EPSP magnitude experienced at the soma (Rhodes, 2006). Several *in vitro* experiments have also reached the conclusion that, in the context of active dendritic integration, the effectiveness of distal inhibition is more potent than previously appreciated (Behabadi et al., 2012; Jadi et al., 2012; Lovett-Barron et al., 2012).

The nonlinear responses of pyramidal neurons are presumed to be affected by inhibition in a location-dependent fashion (Jadi et al., 2012; Lovett-Barron et al., 2012). However, it remains unclear how specific interneuron subtypes affect active dendritic synaptic integration. Naturally, their distinct subcellular targeting is expected to drive varying impacts. Prior investigations have mainly focused on establishing connectivity rules (Jiang et al., 2013; Pfeffer et al., 2013), rather than assessing effects on synaptic integration. *In vivo* studies have assessed interneuron activity and/or examined the effects of manipulations of interneuron activity, where excitatory synaptic input is not under the control of the experimenter (Atallah et al., 2012; Lee et al., 2012; Wilson et al., 2012; Cottam et al., 2013; Seybold et al., 2015; Phillips and Hasenstaub, 2016). Here, we manipulated two of the most prevalent interneuron subtypes with distinct axonal projection patterns: somatostatin-expressing (SST) cells and parvalbumin-expressing (PV) cells. Approximately 60% of PV cell synapses onto layer 2/3 pyramidal cells are found in the perisomatic and proximal dendritic regions (Di Cristo et al., 2004). In contrast, SST cells are biased toward distal regions, sending >90% of their axonal projections to dendrites (Di Cristo et al., 2004; Wang et al., 2004). Using whole-cell recordings of layer 2/3 pyramidal neurons (Cash and Yuste, 1999; Schiller et al., 2000; Ross et al., 2005; Behabadi et al., 2012; Jadi et al., 2012; Bock and Stuart, 2016), in combination with electrical stimulation of excitatory inputs in layer 2/3 and optogenetic activation of interneurons, we report how distinct interneuron subtypes differentially influence active dendritic integration.

## Materials and Methods

### Animals

All procedures involving animals were conducted in accordance with the guidelines and regulations of the US Department of Health and Human Services and approved by the Institutional Animal Care and Use Committee of the University of North Carolina. Transgenic mice that express an improved light-activated cation channelrhodopsin [hChR2/H134R; hereafter called ChR2 (channelrhodopsin-2)] and tdTomato (tdTom) fusion protein in a Cre-dependent fashion (Ai27; catalog #012567, The Jackson Laboratory), were crossed with animals expressing Cre-recombinase under SST promoter (catalog #018973, The Jackson Laboratory; confirmed with histology; Extended Data Fig. 2-1) or PV promoter (catalog #017320, The Jackson Laboratory). Resultant heterozygous animals used in the experiments thus had ChR2 and tdTom expression in either SST or PV cells. Equal numbers of male and female littermates from each genotype were used for all experiments. Mice were housed in a temperature- and humidity-controlled environment on a 12 h light/dark cycle with *ad libitum* access to food and water.

### Slice preparation

Cortical brain slices were dissected from adult transgenic mice ranging in age from postnatal day 30 (P30) to P76. Slices were generated as described previously (Judson et al., 2016). Briefly, mice were anesthetized with pentobarbital sodium (40 mg/kg) and, following the loss of corneal reflex and toe-pinch response, were transcardially perfused with chilled dissection buffer containing the following (in mm): 87 NaCl, 2.5 KCl, 1.25 NaH_2_PO_4_, 26 NaHCO_3_, 75 sucrose, 10 dextrose, 1.3 ascorbic acid, 7 MgCl, and 0.5 CaCl, bubbled with 95% O_2_ and 5% CO_2_. Mice were decapitated, their brains were rapidly removed, and 350-μm-thick coronal slices were cut in chilled dissection buffer using a vibrating microtome (model VT1000S, Leica). Slices were quickly transferred to a holding chamber to recover at 35°C for 20 min in artificial CSF (aCSF) containing the following (in mm): 124 NaCl, 3 KCl, 1.25 NaH_2_PO_4_, 26 NaHCO_3_, 1 MgCl, 2 CaCl, 1.25 ascorbic acid, and 20 dextrose, bubbled with 95% O_2_ and 5% CO_2_. Following recovery, the holding chamber was transferred to room temperature for a minimum of 40 min before slices were used. Recordings were made in a submersion chamber perfused with bubbled aCSF at 2 ml/min with temperature maintained at 33°C. For some experiments, 100 μm aminophosphonovalerate (APV) was added to the aCSF.

### Electrophysiology

Patch-clamp pipettes were pulled from borosilicate glass using a gravity-driven pipette puller (model PC-10, Narishige). Pipette tip resistances ranged from 4.2 to 7.8 MΩ when filled with an internal solution containing the following (in mm): 135 K^+^ gluconate, 4 KCl, 10 HEPES, 10 Na_2_-phosphocreatine, 4 Mg-ATP, 0.3 Na-GTP, 0.025 Alexa Fluor 594, with pH adjusted to 7.25 with KOH, and osmolarity adjusted to ∼295 mmol kg^−1^ with sucrose as needed. Layer 2/3 visual cortex was visualized for whole-cell recording on an upright microscope (Axio Examiner, Zeiss) using infrared differential interference contrast or by fluorescence-based targeting for tdTom^+^ neurons. Neurons were recorded in current-clamp configuration using a patch-clamp amplifier (Multiclamp 700B, Molecular Devices) and pCLAMP 10 software (Molecular Devices). Following an initial pipette seal resistance of ≥1 GΩ, capacitive transients were minimized before breaking into the cell. Input resistance was monitored by test current pulses. Cells were discarded if series resistance was initially >25 MΩ or if either series or input resistance changed by >25% throughout the duration of recording. The bridge was rebalanced as necessary. Layer 2/3 pyramidal cell identity was confirmed by analysis of intrinsic membrane properties, IPSC responses to optogenetic stimulation, firing patterns to depolarizing current steps, and/or the presence of dendritic spines and apical dendrites after being filled with Alexa Fluor 594. Interneuron subtypes were identified by fluorescence, intrinsic membrane properties, response to optogenetic stimuli, and firing response to depolarizing current steps.

**Figure 1.**
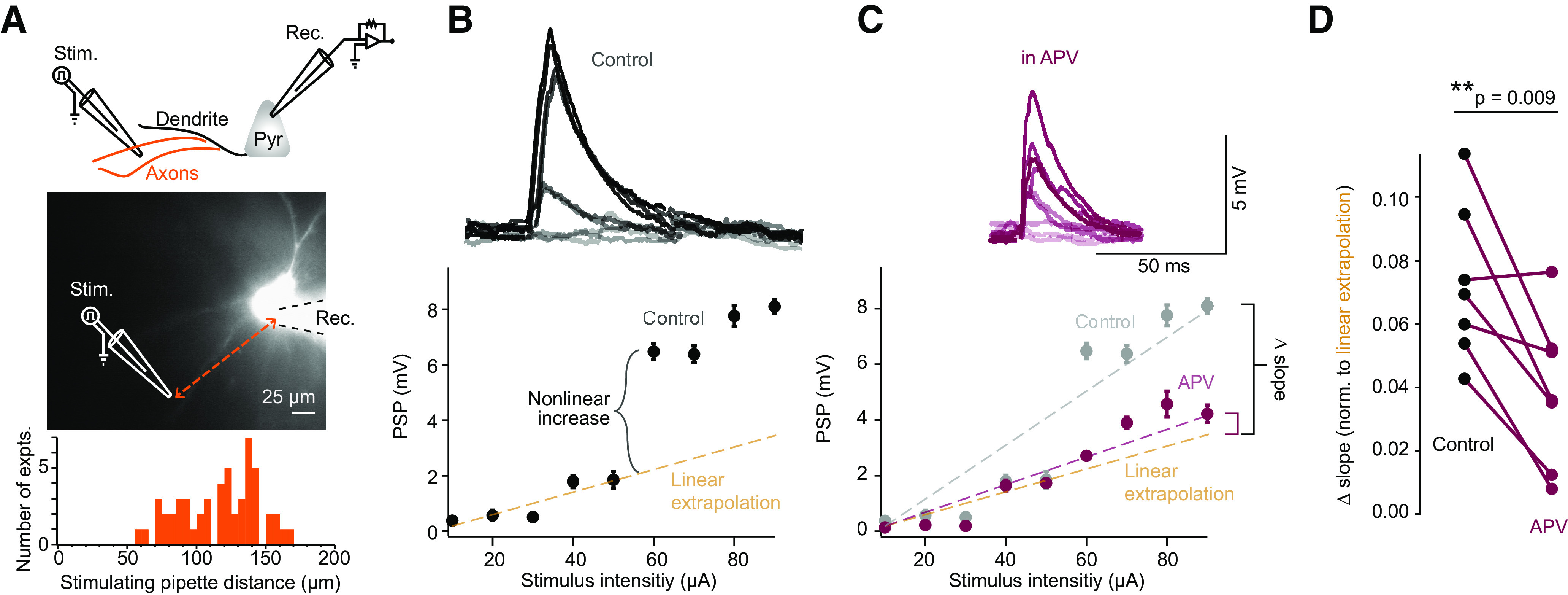
Electrical stimulation of afferent axons in layer 2/3 resulted in NMDAR-dependent dendritic supralinearities. ***A***, Top, Diagram of recording configuration. Middle, Example infrared image of layer 2/3 pyramidal cell filled with fluorescent Alexa Fluor 594 dye. Recording patch pipette is outlined for illustrative purposes. Approximate location of theta glass stimulating pipette, which was placed within layer 2/3, is also indicated. Bottom, Distribution of distance between stimulating pipette and the recorded cell soma. ***B***, Example I–O curve showing suprathreshold excitatory response to linearly increasing stimulus pulses (100 μs duration). Dashed line indicates linear extrapolation of mean PSP values before responses become supralinear. Inset, example voltage trace responses. Error bars indicate the mean ± SEM. The order in which different stimulus intensities were presented did not affect PSP magnitudes (Extended Data Fig. 1-1). One cell exhibited a sublinear response curve (Extended Data Fig. 1-2). A minority of cells exhibited a linear response (Extended Data Fig. 1-3), while a few other cells responded with multiple instances of nonlinear increases in their PSPs (Extended Data Fig. 1-4). ***C***, Same as in ***B*** in the presence of 100 μm APV. Color-coded dashed lines indicate a linear fit of the entire I–O function. ***D***, Change in slope (in millivolts per microampere) for the entire I–O function in control aCSF (*n* = 8) and in the presence of APV (*n* = 8). The application of APV also significantly shortened the duration of PSPs (Extended Data Fig. 1-5). **p ≤ 0.01.

For dendrite-dependent nonlinearity experiments in layer 2/3 pyramidal cells, synaptic stimulation was performed as follows: after achieving a whole-cell recording configuration, the fluorescent signal from the Alexa Fluor 594 was used as a guide to visually place a borosilicate theta-stimulating pipette (World Precision Instruments) filled with aCSF in close proximity (∼5 μm) to the dendritic arbor of the cell, within L2/3. Alternatively, if the dendritic arbor could not be visualized, the stimulating pipette was placed ∼125 μm away from the soma within layer 2/3 (Fig. 1*A*, histogram). Afferent axons from nearby cells could then be electrically stimulated (0.1 ms duration at various stimulus intensities, repeated for five sweeps) to elicit dendritic spikes. The stimulus intensity (SI) value required to produce a somatically detectable postsynaptic potential (PSP) response was cell dependent, and ranged from 20 to 240 μA with a median of 40 μA (mean, 46.363 ± 4.731 μA; *n* = 55). Once a detectable (i.e., ∼0.5 mV) PSP was achieved, the SI value was linearly increased by 10 or 20 μA steps until one of the following three scenarios was achieved: a clearly nonlinear increase in PSPs occurred, after which at least three additional SI values were recorded; the cell began to fire action potentials; or a depolarization of >35 mV occurred. The number of stimulus intensity values used to achieve these criteria range from 8 to 20 with a median of 11. To test for potential confounding effects of linearly increasing the SI value, SI values were presented in decreasing steps in a subset of cells. No differences in PSP values were observed between these trials and trials in which the intensity was linearly increased (Extended Data Fig. 1-1). To evaluate the effect of optogenetic activation of a given interneuron subtype on dendrite-dependent nonlinear increases in PSP values, a 100 ms pulse of 450 nm light was delivered across the surface of the slice via a reflected laser pulse (Techhood Laser). When electrical stimulation of nearby axons was paired with optogenetic stimulation, the electrical pulse was initiated 50 ms after the onset of a 100 ms light pulse. We used a light intensity that evoked reliable spiking responses in SST and PV cells. We used the same intensity for both SST and PV experiments (confirmed by power meter measurement to be 884 mW, which corresponds to 6.25 mW/mm^2^ for the spatial spread of light in our optical system). For these experiments, each SI value was repeated twice per sweep, once under control conditions and then again at the midpoint of the 100 ms light pulse.

10.1523/ENEURO.0235-21.2021.f1-1 Figure 1-1 Order of stimulus intensity presentation did not influence PSP magnitude. ***A***, Diagram of recording configuration. ***B***, Example PSP response to linearly increasing (black) or decreasing (green) stimulus intensities. Inset, sample voltage traces. Download Figure 1-1, EPS file.

**Figure 2.**
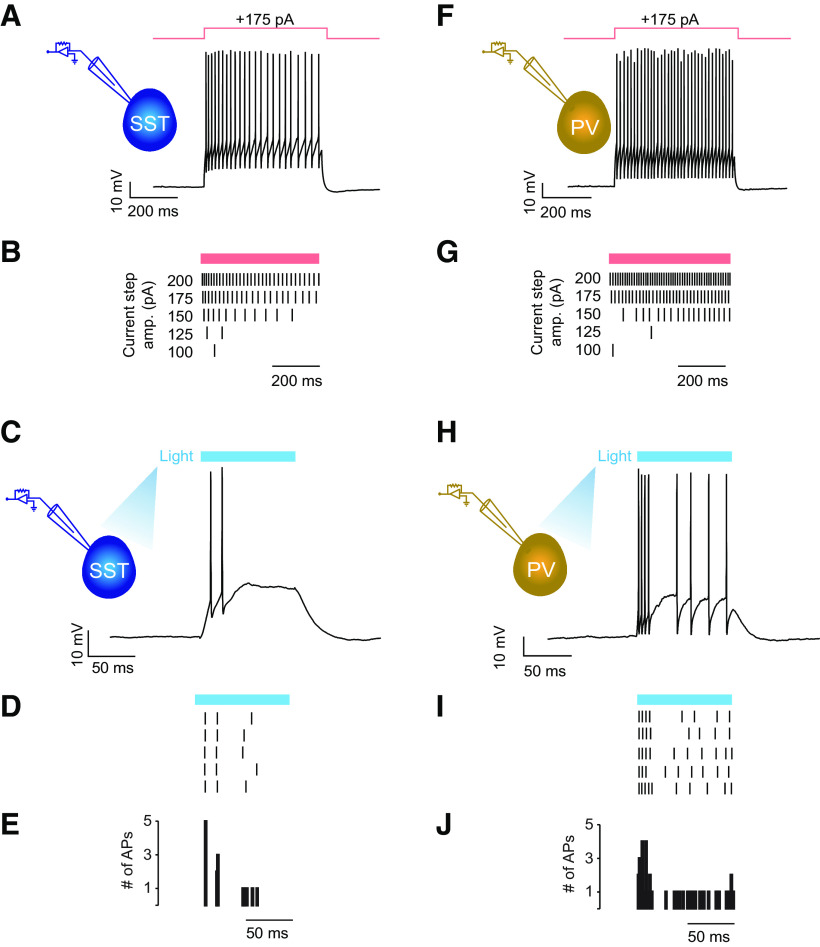
ChR2-expressing SST and PV neurons responded reliably to light activation. ***A***, Top left, Diagram indicating the recording configuration for a tdTom-expressing and ChR2-expressing SST neuron. Immunohistochemistry confirmed that the majority of tdTom^+^/ChR2^+^ cells were SST^+^ (Extended Data Fig. 2-1). Right, Example cell response to a 500-ms-long current step of +175 pA showing the accommodation expected for SST neurons. ***B***, Spike raster of the response of the same cell to increasing steps of depolarizing current, again showing accommodation. ***C***, Response of the cell to a 100-ms-long light pulse of 450 nm light. ***D***, Spike raster for the response of a cell to the light pulse. ***E***, Peristimulus time histogram over trials. ***F***, Same as ***A*** for a tdTom-expressing and ChR2-expressing PV neuron. As expected for PV neurons, the spike response does not accommodate (in contrast to SST neurons). ***G***, Spike raster of the response of the same cell to increasing steps of depolarizing current. ***H***, Response of a cell to a 100-ms-long light pulse of 450 nm light. ***I***, Spike raster for the response of a cell to the light pulse. ***J***, Peristimulus time histogram over trials. Blocking inhibition with PTX resulted in an increase of spike response to light in SST cells but not in PV cells (Extended Data Fig. 2-2).

10.1523/ENEURO.0235-21.2021.f1-2 Figure 1-2 Quantification of a pyramidal cell that exhibited a sublinear response to electrical stimulation. ***A***, Top, Cartoon schematic of recording configuration. Bottom, Example infrared image of layer 2/3 pyramidal cell filled with fluorescent Alexa Fluor 594 dye. ***B***, I–O plot showing the sublinear response to linearly increasing current stimulations in control aCSF (black) or aCSF containing 100 μm APV (purple). Download Figure 1-2, EPS file.

**Figure 3.**
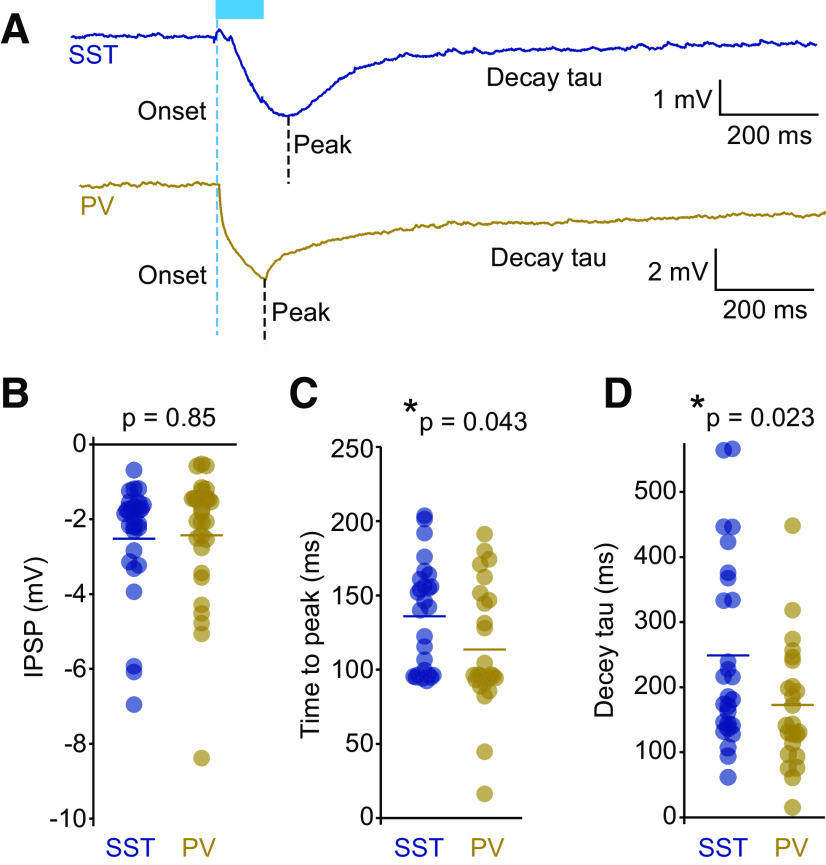
Similar IPSPs were recorded in pyramidal neurons when ChR2-expressing SST or PV neurons were activated with a light pulse. ***A***, Example traces of IPSP responses in recorded pyramidal cells following optogenetic activation of either SST (blue; *n* = 27) or PV (gold; *n* = 28) cells. ***B***, IPSP peak magnitudes were similar in both experiments. ***C***, ***D***, SST cell-mediated evoked IPSPs (***C***) peaked later and decayed with longer time constants than PV cell-mediated evoked IPSPs (***D***). **p ≤ 0.05.

10.1523/ENEURO.0235-21.2021.f1-3 Figure 1-3 A minority of cells did not exhibit a nonlinear response to stimulation. ***A***, Example linear I–O plot to increasing levels of electrical stimulation in the absence (black) or presence (blue) of optogenetic activation of SST cells. Inset, Sample voltage traces at maximal stimulus intensity. Dashed line (purple) indicates linear extrapolation. Error bars indicate the mean ± SEM. ***B***, Same as in ***A***, with PV cell activation in gold. Download Figure 1-3, EPS file.

**Figure 4.**
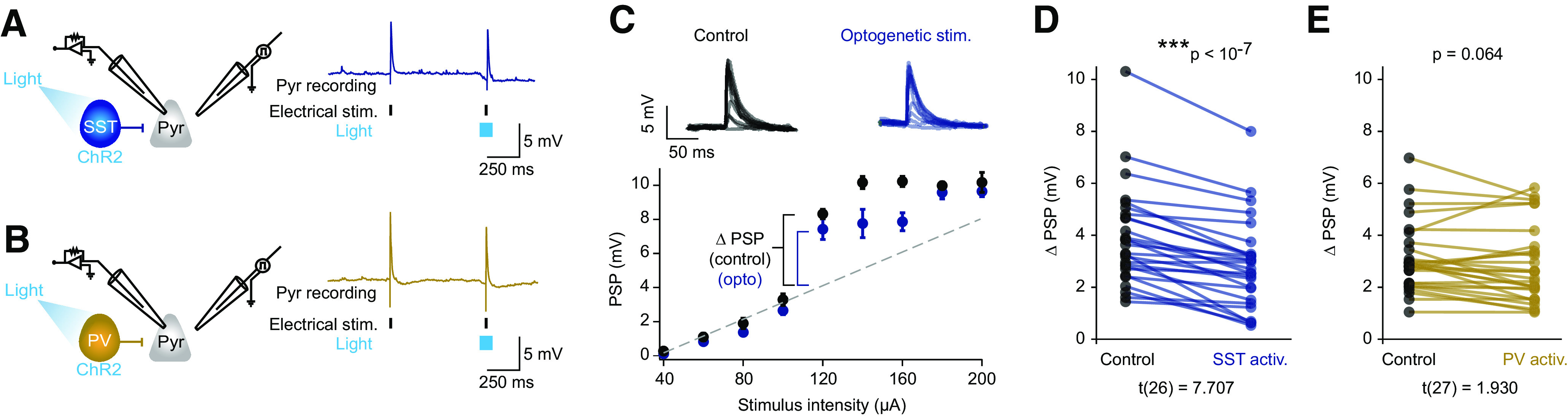
SST cell activation reduced the magnitude of nonlinear responses. ***A***, Left, Diagram depicting recording and optogenetic activation of SST cells. Right, Example pyramidal cell response to electrical stimulation (hash marks) and 100 ms optogenetic stimulation of SST cells (light blue). ***B***, Same as in ***A***, with the activation of PV cells in gold. ***C***, Top, Example PSP response to electrical stimulation alone (black) or in combination with the optogenetic activation of SST cells (blue). Bottom, Example I–O curve during control stimulation (black) and optogenetic activation of SST cells (blue). Dashed line indicates linear extrapolation of PSP amplitudes based on the first four SIs. Error bars indicate the mean ± SEM. The Δ PSP indicated is the supralinear jump in PSP size in the I–O curve. ***D***, Comparison of PSP jump under control conditions (black) and during optogenetic activation of SST cells (blue; *n* = 27). ***E***, Same as in ***D*** with PV cell activation in gold (*n* = 28). The I–O curve was linearized by optogenetic activation of interneurons in a minority subset of cells (6 of 27 SST cells; 2 of 28 PV cells; Extended Data Fig. 4-1). ***p ≤ 0.001.

10.1523/ENEURO.0235-21.2021.f1-4 Figure 1-4 A minority of cells exhibited multiple instances of nonlinear increases in response to stimulation. **A**, Top, Diagram depicting recording and optogenetic activation of an SST cell. Bottom, Example I–O curve with increasing electrical stimulation in the absence (black) or presence (blue) of optogenetic activation of SST cells (blue). ***B***, Same as ***A***, with the activation of PV cells in gold. Download Figure 1-4, EPS file.

**Figure 5.**
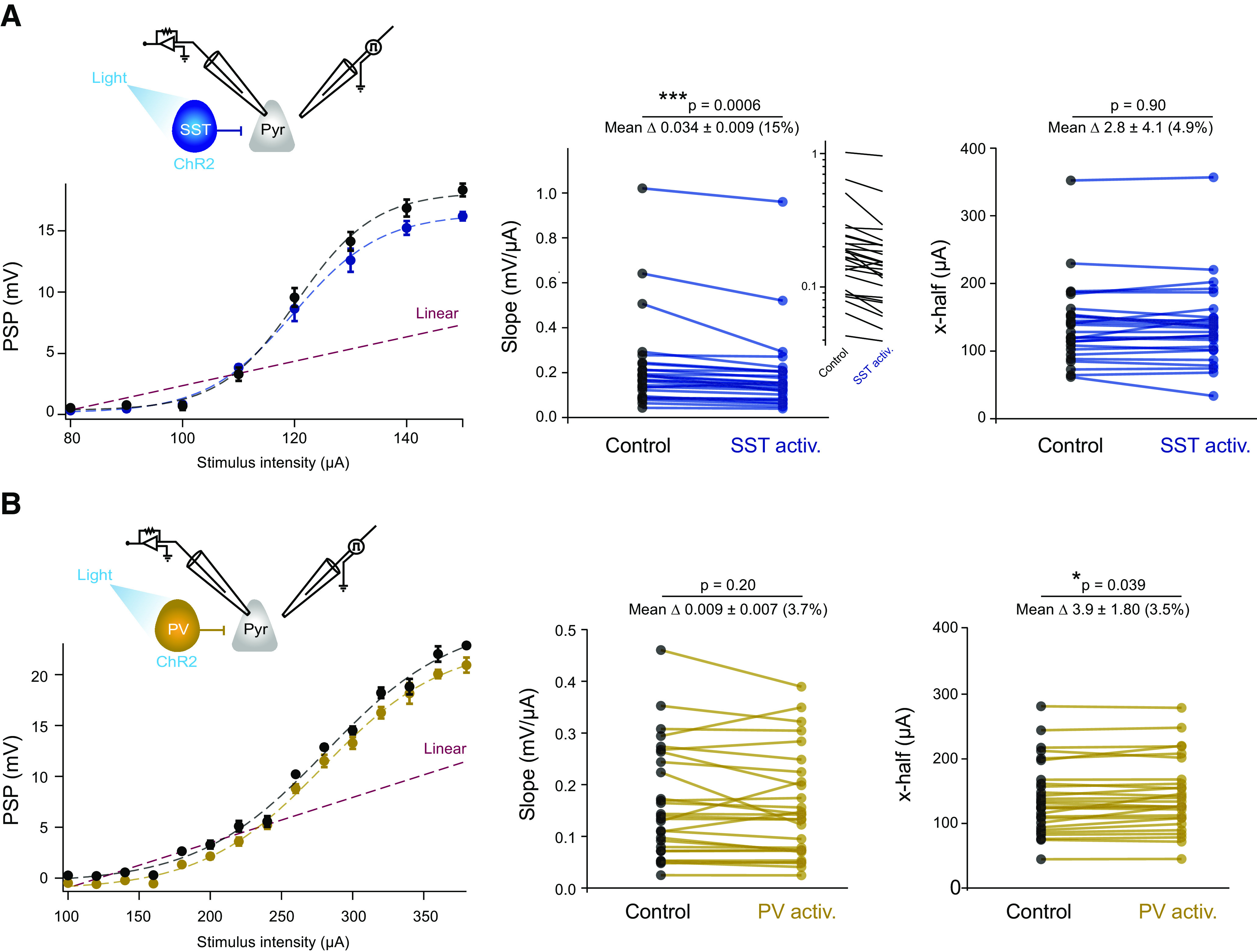
SST cells predominantly mediated divisive gain control, while PV cell activation resulted in subtractive inhibition. ***A***, Left, Example I–O, fitted with a sigmoidal curve, in the absence (black) or presence (blue) of optogenetic activation of SST cells. Dashed line indicates linear extrapolation from first four data points. Error bars indicate the mean ± SEM. Middle, Comparison of the slope of sigmoidal fit during the control and optogenetic activation of SST cells. A logarithmic plot of the same data is shown in the inset. Right, Comparison of the *x*-half (offset) of sigmoidal fit during control and optogenetic activation of SST cells (*n* = 27). ***B***, Same as in ***A***, with the activation of PV cells in gold (*n* = 28). *p ≤ 0.05, ***p ≤ 0.001.

10.1523/ENEURO.0235-21.2021.f1-5 Figure 1-5 NMDA receptor blockade with APV affected PSP kinetics. ***A***, Top, Example voltage traces. Bottom, Comparison of the FWHM of induced PSP at maximal stimulus intensities in control aCSF (black) or aCSF containing 100 μm APV (purple). ***B***, Same as in ***A*** with the comparison of the decay constant tau in control (black) and APV-containing (purple) aCSF (*n* = 8). Download Figure 1-5, EPS file.

### Immunofluorescence

Animals were anesthetized with a mixture of ketamine (100 mg/kg) and xylazine (15 mg/kg), and were intracardially perfused with PBS followed by 4% paraformaldehyde. After fixing overnight, 50 μm sections were cut and rocked in a blocking buffer containing 0.02% sodium azide, 0.03% bovine serum albumin, 0.05% goat serum, and 0.2% Triton X-100 in 250 ml of PBS for 1 h. Primary antibody solutions were prepared in PBS using rabbit anti-RFP (1:400; catalog #600–401-379, Rockland) and rat anti-SST (1:400; catalog #MAB345, Millipore Sigma) antibodies. Primary antibody solutions were added to slices and incubated overnight at 4°C. Sections were then washed in blocking buffer at room temperature 3× for 15 min each and incubated in secondary antibody solutions containing goat anti-rabbit (1:500; catalog #A10520, Thermo Fisher Scientific) and goat anti-rat (1:500; catalog #A11006, Thermo Fisher Scientific) for 2 h at room temperature. Sections were then washed in blocking buffer at room temperature 2× for 15 min then once more in PBS containing 0.1% Tween 20 for 15 min. DAPI staining (1:1000 dilution in PBS) occurred at room temperature for 15 min followed by a final wash in PBS at room temperature for 15 min. Sections were then mounted and imaged.

### Analysis

Recording data were analyzed using custom scripts for IGOR Pro analysis software (WaveMetrics), including event detection and analysis routines written by T. Ishikawa (Jikei University, Tokyo, Japan). To ascertain whether electrically induced nonlinear responses were dependent on NMDAR activation, input–output (I–O) plots of PSP versus SI values were fit to linear regression (*y = a + bx)* for cells in control aCSF and in aCSF containing 100 μm APV, and the slopes of the linear fit were compared between the two conditions. One cell exhibited a sublinear I–O curve in both control and APV containing aCSF and was excluded from further analysis (Extended Data Fig. 1-2). Cells were analyzed for nonlinearity by comparing mean somatic PSP responses to a linear extrapolation of previous mean values to determine the nonlinearity relative to linear extrapolation ratio (Behabadi et al., 2012). Briefly, the SI values with the largest difference in PSP responses (i.e., largest Δ value; SI_supralinear_) were identified from I–O plots. All mean PSP responses leading up to the identified SI were then linearly fit. The experimentally derived PSP for the SI_supralinear_ was then compared with the expected value based on the linear extrapolation. Cells that had at least one experimental PSP value that exceeded the expected value by one-third were considered to display a nonlinear response profile, while cells that did not were considered linear and were excluded from analysis (a total of 14 of 69 cells were linear; Extended Data Fig. 1-3, example cells). If a cell displayed multiple points of nonlinearity, the first instance was considered for analysis (Extended Data Fig 1-4, example cells with multiple nonlinear events). To determine how SST and PV cell activation affected the magnitude of the dendritic nonlinearity, the difference between the experimental PSP values and the linear extrapolation at SI_supralinear_ was compared under a control condition and during optogenetic activation. To assess changes in gain and offset, the entire I–O curves under control and optogenetic conditions were fit to a sigmoid: base+{max/(1+exp((x-half−x)/rate)}, where base and max are the baseline and maximal responses, respectively, and rate determines the slope parameter (Lovett-Barron et al., 2012). From this fitting, we were able to calculate the degree of separation along the *x*-axis between control and optogenetic conditions using the *x*-half parameter. Furthermore, changes in slope because of optogenetic activation could be assessed by comparing the peak of the first derivative of the sigmoidal fit during control and optogenetic conditions.

### Statistics

Unless otherwise stated, all measurements are presented as the mean ± SEM. Randomization and experimental blindness were not used for electrophysiology data as each cell serves as an internal control (e.g., PSP value during control stimulation or in the presence of optogenetic activation of interneuron subtypes). Statistical differences between control conditions and during optogenetic activation of interneuron subtypes were assessed by paired *t* tests with α = 0.05.

## Results

### Electrical stimulation of afferent axons results in NMDAR-dependent supralinear integration

We made whole-cell current-clamp recordings from layer 2/3 pyramidal cells in slices of mouse visual cortex. To activate nonlinear mechanisms on dendrites, we electrically stimulated nearby axons using a theta stimulating pipette placed within layer 2/3 (Fig. 1*A*). The stimulus pipette was typically over 100 μm away from the soma and was in the direction lateral and basal from the cell body. Thus, the stimulated inputs were likely axons in layer 2/3. These axons likely synapsed on both basal and apical dendrites. Brief (0.1 ms) constant current pulses were sufficient to elicit PSPs at the soma. Approximately 80% of the pyramidal cells tested (55 of 69 cells) exhibited evidence of supralinear synaptic integration above a certain stimulus threshold, similar to those reported in prior studies (Schiller et al., 2000; Branco et al., 2010; Fig. 1*B*, Extended Data Fig. 1-3, examples of the few linear cells). In response to increasing SIs, PSPs increased linearly at first. However, at a threshold SI (which varied from cell to cell, but was reproducible within a given cell), the PSPs increased supralinearly, likely because of the recruitment of active (i.e., voltage-gated) mechanisms (Fig. 1*B*).

To identify the voltage-dependent channels contributing to the nonlinear response, we blocked NMDA receptors with the competitive antagonist APV (Fig. 1*C*). NMDA receptors are a major active component linking synaptic input to supralinear PSPs (Schiller et al., 2000; Branco et al., 2010). Because the inactivation time constant of NMDA receptors is ∼10-fold greater than that of AMPA receptors, PSPs should be shorter in duration if NMDA receptors are blocked. Indeed, bath application of APV reduced the durations of PSPs [Extended Data Fig. 1-5; mean full-width at half-maximal (FWHM) control = 20.9 ± 2.4 ms; mean FWHM APV = 12.0 ± 1.7 ms; *t*_(7)_ = 3.066, *p *=* *0.009, *n* = 8, paired *t* test; mean tau control = 26.3 ± 2.9 ms; mean tau APV = 15.1 ± 2.2 ms, *t*_(7)_ = 6.018, *p *=* *0.005, *n* = 8, paired *t* test]. Blocking NMDA receptors also resulted in significant reductions in the slopes of the I–O curves (Fig. 1*D*), bringing the mean PSP levels closer to the linear trajectory extrapolated from the lower stimulus intensities (46.6% reduction in slope; mean slope control = 0.06 ± 0.01 mV/μA; mean slope APV = 0.03 ± 0.01 mV/μA; *t*_(6)_ = −4.256, *p *=* *0.009, *n* = 7, paired *t* test). Blocking NMDA receptors did not result in a completely linear I–O curve in most cells, however, suggesting residual contributions from voltage-gated channels (e.g., voltage-gated Na^+^ and voltage-gated Ca^2+^ channels) to dendritic nonlinearities (Smith et al., 2013). Overall, I–O curves were made more linear, and the slope was reduced by half after NMDA receptors were blocked. Thus, brief electrical excitation of axons recruits NMDA receptor-dependent active mechanisms on the dendrites of layer 2/3 visual cortex neurons, and this can be measured as nonlinear increases in the SI/PSP I–O relationship.

### Activation of SST but not PV cells decreases enhancement of PSP amplitudes by active dendrites

To investigate the roles that SST and PV cells play in synaptic integration, we generated transgenic mice that would allow us to specifically manipulate the activity of each subtype via optogenetics. We crossed mice that expressed Cre-recombinase in either SST or PV cells with mice that expressed the light-activated cation channel ChR2 and tdTom as a fusion protein in a Cre-dependent fashion. The resultant mice thus expressed ChR2/tdTom in either SST cells (SST-Cre^+^/ChR2^+^/tdTom^+^) or PV cells (PV-Cre^+^/ChR2^+^/tdTom^+^). We confirmed that ChR2-expressing SST cells exhibited accommodating spike responses (Fanselow et al., 2008) to current injections and fired spikes in response to blue light (Fig. 2*A–E*). Similarly, we confirmed that ChR2-expressing PV cells exhibited nonaccommodating spike responses (Fanselow et al., 2008) to current injections and fired spikes in response to blue light (Fig. 2*F–J*). Blocking inhibition with picrotoxin (PTX) resulted in an increase of spike output from SST neurons, but not from PV neurons (Extended Data Fig. 2-2).

10.1523/ENEURO.0235-21.2021.f2-1 Figure 2-1 The majority of tdTom^+^/ChR2^+^ cells were SST^+^. ***A***, Left, Representative image of coronal section of mouse visual cortex stained for somatostatin. Middle left, Enlarged section of leftmost image showing cells positive for somatostatin expression. Middle right, Same region, showing cells positive for tdTomato expression. Right, Merged image. ***B***, Quantification of cell expression, mean ± SEM (six sections, two mice). Download Figure 2-1, EPS file.

10.1523/ENEURO.0235-21.2021.f2-2 Figure 2-2 Spike responses in SST cells were increased by blocking inhibition using PTX. ***A***, Diagram indicating recording configuration and PTX blocking of inhibition. ***B***, Top, Example responses of SST cell to 10 Hz trains of 450 nm light pulses (20 ms pulse width) in control aCSF (black) and aCSF containing 50 μm PTX (blue). Bottom, Comparison of spike probability to all pulses in a 10 Hz train of light in control aCSF and PTX (*n* = 14). ***C***, Same as ***B*** showing SST cell response to 20 Hz trains of 450 nm light pulses (*n* = 13). ***D***, Same as ***A*** with recording/activation of PV cells in gold. ***E***, Same as ***B*** with recording/activation of PV cells in gold (*n* = 10). ***F***, Same as in ***E***, showing PV cell response to 20 Hz trains of 450 nm light pulses in gold (*n* = 10). Download Figure 2-2, EPS file.

Using these mice, we made whole-cell recordings from pyramidal neurons as before in acute visual cortex slices. Optogenetically evoked IPSP amplitudes recorded from the pyramidal neurons were similar for both SST and PV cell activations (Fig. 3*A*,*B*; SST: mean = −2.518 ± 0.299 mV, *n* = 27; PV: mean = −2.432 ± 0.325 mV, *n* = 28; *t*_(54)_ = 0.195, *p *= 0.846, paired *t* test). However, they exhibited different time courses, with the SST-driven IPSPs displaying a significantly longer time-to-peak (Fig. 3*C*; SST mean = 135.9 ± 7.1 ms, *n* = 27; PV mean = 113.5 ± 8.1 ms, *n* = 28; *t*_(54)_ = −2.08, *p *=* *0.043, paired *t* test) and slower decay time compared with IPSPs driven by PV cell activation (Fig. 3*D*; SST mean = 248.9 ± 27.7 ms, *n* = 27; PV mean = 172.8 ± 17.8 ms, *n* = 28; *t*_(54)_ = −2.35, *p *=* *0.023, paired *t* test). This approach provides a way to activate inhibitory inputs from PV or SST neurons and determine the resulting PSPs in pyramidal neurons.

Next, we examined the degree to which activating SST or PV neurons affected the I–O curves. Specifically, we were interested in whether the supralinear responses would be preserved during optogenetic activation. To do so, we used an interleaved testing approach. In each sweep for a particular electrical stimulus amplitude of the I–O curve, we delivered the stimulus once without any optogenetic manipulation followed by another while optogenetically stimulating SST or PV interneurons (Fig. 4*A*,*B*). This was repeated at least five times for each stimulus intensity.

10.1523/ENEURO.0235-21.2021.f4-1 Figure 4-1 A minority of cells exhibited nonlinear increases during control stimulation that were linearized during optogenetic stimulation. ***A***, Top, Diagram depicting the recording and optogenetic activation of SST cells. Bottom, Example input–output trace during control stimulations (black) or stimulations during optogenetic activation of SST cells (blue). Dashed line (purple) indicates linear extrapolation. ***B***, Same as ***A*** with activation of PV cells in gold. Download Figure 4-1, EPS file.

Of the pyramidal neurons that exhibited a supralinearity in their I–O curves (55 of 69 neurons), the I–O curve was linearized by optogenetic activation of interneurons in a minority subset (8 of 55 neurons). This occurred more commonly with SST neuron activation (6 of 27 neurons) than with PV neuron activation (2 of 28 neurons; Extended Data Fig. 4-1). Thus, although neither subtype consistently eliminated the nonlinearity, SST neurons more frequently linearized responses than did PV neurons. In the majority of cells (47 of 55 cells) that exhibited a nonlinearity in their I–O curves under control conditions, the optogenetic activation of interneurons altered, but did not eliminate, the nonlinear response. To quantify the effects of SST and PV cells on the I–O curves, we determined the SI value at which responses became supralinear (for further details, see Materials and Methods) and measured the difference between the experimentally observed PSP and the expected PSP based on a linear extrapolation at that SI value (Fig. 4*C*). We found that optogenetic activation of SST neurons resulted in a significant reduction in the magnitude of the nonlinear response compared with control conditions (Fig. 4*D*; control mean = 3.9 ± 0.4 mV; optogenetic mean = 2.9 ± 0.3 mV; *t*_(26)_ = 7.707, *p *=* *3.9 * 10^−8^, *n* = 27, paired *t* test). By contrast, activating PV neurons during electrical stimulation did not significantly alter the response (Fig. 4*E*; control mean = 3.4 ± 0.3 mV; optogenetic mean = 3.0 ± 0.3 mV; *t*_(27)_ = 1.930, *p *=* *0.064, *n* = 28, paired *t* test). For those cells whose response remained nonlinear, the SI value at which the responses became supralinear did not change on optogenetic stimulation for either cell types (SST control mean = 119.09 ± 12.11, optogenetic mean = 119.09 ± 12.11; *t*_(21)_ = NaN, *p* = 1, *n* = 22, paired *t* test; PV control mean = 133.846 ± 11.90, optogenetic mean = 132.308 ± 11.0; *t*_(25)_ = 0.527, *p* = 0.603, *n* = 27, paired *t* test). Thus, optogenetic activation of SST neurons, more so than PV neurons, suppressed supralinear PSPs in pyramidal neurons in layer 2/3 without affecting the gross electrical stimulus intensity threshold for supralinearity.

### SST cells mediate predominantly divisive gain control

To further quantify how SST and PV neurons differentially affected the overall I–O functions, we fit the data using sigmoid curves (for further details, see Materials and Methods). Activation of SST cells reduced the slope of the sigmoid fit compared with control (Fig. 5*A*; mean slope control = 0.22 ± 0.04 mV/μA, mean slope optogenetic = 0.19 ± 0.04 mV/μA; *t*_(26)_ = 3.904, *p *=* *0.0006, *n* = 27, paired *t* test). This effect is qualitatively similar to the one quantified earlier (Fig. 4*E*). Of note, SST activation had no effect on the offset (*x*-half) of the curve fits, which further confirmed that there was no shift in the threshold for supralinear PSPs (Fig. 5*A*; mean offset control = 120.0 ± 17.5 μA, mean offset optogenetic = 118.5 ± 18.0 μA; *t*_(26)_ = 0.132, *p *=* *0.896, *n* = 27, paired *t* test).

By contrast, the activation of PV cells slightly but significantly increased the offset of the I–O curve fits (Fig. 5*B*; mean offset control = 139.0 ± 10.5 μA, mean offset optogenetic = 142.9 ± 10.4 μA; *t*_(27)_ = −2.167, *p *=* *0.039, *n* = 28 paired *t* test), but had little effect on the slope (Fig. 5*B*; mean slope control = 0.16 ± 0.02 mV/μA, mean slope optogenetic = 0.16 ± 0.01 mV/μA, *t*_(27)_ = 1.329, *p *=* *0.195, *n* = 28, paired *t* test). This small change in the offset was driven by the two cells whose responses were linearized by PV activation, as the offset was no longer significantly different when these cells were excluded although the trend remained the same (*t*_(25)_ = −1.919, *p *=* *0.0665). Excluding the six cells that were linearized by SST activation did not affect the *x*-half (*t*_(21)_ = 0.1328, *p *=* *0.89, *n* = 22). Taken as a whole, these results demonstrate that SST cells have a greater effect than PV cells on voltage-dependent synaptic integration. SST cells mediate a predominantly divisive form of inhibitory gain control during active synaptic integration, while PV cells appear to contribute modest subtractive inhibition.

## Discussion

Here, we examined inhibitory influences on nonlinear dendritic synaptic integration in the dendrites of L2/3 neurons. Optogenetic manipulation of the two largest interneuron subtypes, SST and PV cells, revealed their distinct inhibitory effects on nonlinear synaptic integration of layer 2/3 inputs. Activating SST cells reduced the magnitude of the somatic depolarization during nonlinear synaptic integration (Fig. 4*D*). By contrast, activating PV cells had only modest effects (Fig. 4*E*). These results support the hypothesis that SST cells, with their more dendrite-biased axonal projection patterns, have a stronger influence over nonlinear integration than PV cells. However, SST cell activation did not shift the threshold level of synaptic input required to activate supralinear response. Thus, SST cells do not regulate the recruitment of voltage-dependent active mechanisms, but modulate the amplitude of the resulting postsynaptic depolarization seen in the soma, placing them downstream of the dendritic mechanism for the nonlinear enhancement of the synaptic inputs, namely dendritic spikes (Smith et al., 2013).

The location of inhibition relative to excitation plays a critical role in synaptic integration (Koch et al., 1983). In passive dendritic trees, inhibition is most effective at modulating excitatory conductances when inhibitory sources are positioned proximal to the site of excitation (Koch et al., 1983; Vu and Krasne, 1992; Liu, 2004; Hao et al., 2009). In the presence of active dendritic mechanisms, the location dependence of inhibition is still strong, but with added complexity. It has been previously shown that in layer 5 pyramidal neurons, focal GABA iontophoresis targeting perisomatic areas during nonlinear responses to glutamate uncaging results in a reduction in the overall magnitude of supralinear responses, while GABA iontophoresis onto basal dendrites leads to a shift in the stimulus laser intensity threshold for supralinear responses (Jadi et al., 2012). Once the increased threshold is reached, however, the magnitude of the somatic depolarizations remains comparable. These reports support the notion of distinct computational roles for proximal and distal inhibition.

Although SST and PV cells have relatively distinct projection patterns, the differences are more subtle than the contrast that can be achieved with local GABA iontophoresis, as used in the aforementioned work (Jadi et al., 2012). In fact, in terms of shear anatomic numbers, PV cell inputs outnumber SST cell inputs on dendrites of layer 2/3 pyramidal neurons by twofold (Kuljis et al., 2019). However, the distribution patterns of the dendritic synapses differ for each subtype. Although PV cell inputs are seen throughout the length of the dendritic tree, they are noticeably concentrated at the primary dendrites with a decline in the higher-order dendrites, whereas SST cell inputs are found on secondary and higher-order dendrites but are absent from the primary dendrites. In line with this distribution pattern, our results show that, on average, SST cell inputs were distal relative to PV cell inputs. Peak IPSP responses resulting from the stimulation of SST cells were significantly later in arriving at the soma than IPSPs evoked from stimulating PV cells (Fig. 3*C*), implying a more distal origin. Despite this, our results differed markedly from the model-based predictions or iontophoresis studies (Jadi et al., 2012), prompting a re-evaluation of physiological roles for SST and PV cells, particularly in the context of nonlinear synaptic integration, at least in L2/3 neurons.

We found that SST cell-mediated inhibition functions both as a restrictor on the absolute charge conveyed to the soma and as a gain modulator, altering the slope of the I–O curve. The divisive effect of activating SST cells reduced both the slope of the I–O plots (Fig. 5*A*) and the magnitude of active dendrite-dependent PSPs (the first nonlinearity step; Fig. 4*C*,*D*) measured at the soma. The inhibition mediated by PV cells had only modest effects on the offset of the I–O curves (Fig. 5*B*). Thus, our findings are more in line with those of a previous study on CA1 hippocampal pyramidal cells, in which a combination of two-photon glutamate uncaging and one-photon GABA uncaging demonstrated that apical dendritic inhibition was more effective than somatic inhibition at shunting nonlinear dendritic responses (Lovett-Barron et al., 2012). Similarly in our study, SST cell-mediated inhibitory inputs (putatively distal relative to PV cell inputs) suppressed supralinear responses recorded at the soma, without affecting the stimulus input threshold for the nonlinear step. At least a couple of factors could account for this observation. First, the original magnitude of IPSPs at synaptic locations may vary between the two subtypes. SST cell-mediated IPSPs took longer to reach peak magnitude than PV cell-mediated IPSPs, indicating that SST cell inputs were more distal. However, the IPSPs recorded at the soma were similar in magnitude for both SST and PV cell activation (Fig. 3*B*). Because of the attenuation of charge during propagation, this implies that the IPSPs experienced at the dendrites were likely greater during SST cell stimulation than PV cell stimulation. If the majority of SST cell inputs were both larger and more distal than PV cell inputs, yet proximal relative to some of the excitatory active synaptic events, this could explain how the nonlinear response was reduced in magnitude in response to SST but not PV cell stimulation, and account for the lack of effect on the initiation of dendritic nonlinearities. Second, the spreading of SST cell-mediated inhibitory current originating in distal dendrites may provide a more effective long-range shunt than the proximal inhibition by PV cells, because of the soma acting as a current sink (Gidon and Segev, 2012).

It is important to note some of the limitations to our study. The first concerns the method used to induce dendritic nonlinearities. The use of electrical stimulation of presynaptic axons is realistic in that it uses physiological synapses likely located at multiple dendritic locations (rather than the uncaging of glutamate at a spatial cluster of dendritic spines that may not be simultaneously activated under physiological conditions). However, it also limits our ability to spatially control the location of synaptic excitation relative to inhibition. We targeted electrical stimulation to distal regions of the dendritic arbor of the recorded cell within L2/3 (Fig. 1*A*), the site for dendritic spike generation in layer 2/3 pyramidal cells of the visual cortex (Smith et al., 2013). It is possible that *in vivo* patterns of excitation and inhibition have a precise architecture (Rossi et al., 2019) that our experiments failed to recreate. In addition, it is also possible that inhibitory axons impinging onto the distal dendrites were activated along with excitatory axons during electrical stimulation, altering the baseline inhibitory activity. This would mean the measured effects would be an underestimate, because of the baseline condition involving nonzero amounts of inhibitory input. The second limitation is that the optogenetic stimulation activated a large population of interneurons (few if any inhibitory neurons failed to respond to the light) that may not be simultaneously active under physiological conditions, and thus the condition may be considered an upper limit case. Lower levels of interneuron activation would be expected to yield smaller changes than those reported here. Relatedly, it should be noted that the level of activation of PV and SST neurons was matched in one way (IPSP amplitude as recorded somatically in pyramidal neurons; Fig. 3), but it is not feasible to precisely match optogenetic control over PV and SST cells in all ways; nor is it simple to define what constitutes a “match.” We settled on the optogenetic light intensity that would reliably elicit spike responses in SST cells and used this same intensity for both SST and PV cell experiments (6.25 mW/mm^2^). The light pulse elicited fewer spikes in SST compared with PV cells, and the more persistent activation of PV cells was evident in the PV-IPSP shape, which exhibited a noticeably sharp offset aligned with the offset of the light stimulation. The SST-IPSP by contrast exhibited a more gradual offset that did not align with the light offset, likely because of the fewer number of evoked spikes followed by response accommodation (Fig. 2). Despite these differences in their somatic spike responses to the optogenetic stimulation, the amplitudes of PV-IPSPs and SST-IPSPs matched well (Fig. 3*B*). On the one hand, this “matched” optogenetically induced IPSP size recorded at the soma may give SST cells an unfair advantage as these inhibitory inputs may be larger at the distal dendrites where SST inputs are. On the other hand, however, PV cells may not be as disadvantaged because the size of electrically induced EPSPs should be significantly reduced by the time they reach the perisomatic area where PV inputs may dominate. The third limitation is that the relative timing between excitation and optogenetically manipulated inhibition did not precisely recreate what occurs *in vivo*. SST and PV cells exhibit different stimulus response properties *in vivo*, with SST cells being more orientation tuned than PV cells, and responding to stimuli with a greater delay (Ma et al., 2010). Whether and how the differential pattern and timing of activation of these two subtypes of interneurons may help shape the synaptic integration and output from the target pyramidal neuron (e.g., as in the model of Barlow and Levick, 1965) remains to be seen *in vivo*. Overall, these limitations are important to consider but leave the qualitative results we report here intact.

In summary, we find that the roles of SST and PV cell-mediated inhibition do not map neatly onto the roles suggested by prior work for dendritic and somatic targeted inhibition. Our results demonstrated that SST cell-mediated inhibition reduces the amplitude of somatic PSPs during active synaptic integration, and PV cell-mediated inhibition does not. More importantly, neither SST nor PV cell activation caused substantial changes in the input threshold for the recruitment of nonlinear mechanisms. Of note, since SST cells inhibit PV cells (Cottam et al., 2013), *in vivo* SST cell activity could have multilayered effects with a combination of divisive suppression of supralinear dendritic response and disinhibition of the perisomatic compartment (Seybold et al., 2015). Together, the impact of inhibition on active synaptic integration may be an analog modulation, rather than a digital on/off switch. When spiking thresholds are taken into account, such modulatory effects can generate diverse signal outputs (Seybold et al., 2015).

## Synthesis

Reviewing Editor: Matthew Grubb, King’s College London Decisions are customarily a result of the Reviewing Editor and the peer reviewers coming together and discussing their recommendations until a consensus is reached. When revisions are invited, a fact-based synthesis statement explaining their decision and outlining what is needed to prepare a revision will be listed below. The following reviewer(s) agreed to reveal their identity: Scott Cruikshank. Note: If this manuscript was transferred from JNeurosci and a decision was made to accept the manuscript without peer review, a brief statement to this effect will instead be what is listed below.

Both Reviewers’ were extremely positive about the revised manuscript. Reviewer 1 made some reasonable and pertinent requests based on their previous comments on the manuscript, so I would like you to address these before I can accept it. In particular, it would seem like the most straightforward option is to take Reviewer 1’s suggestion of adding to your Discussion section the argument you present in your rebuttal letter concerning matching inhibitory output between different cell types. Please also add the requested shift measurement (see comments below).

Reviewers’ comments

Reviewer 1:

This is a revised manuscript that I previously reviewed for the Journal of Neuroscience. I was generally enthusiastic about the previous version and the authors have made a good-faith effort to address the majority of my comments in this version. However, I do not think that the first major issue raised in my previous review has been adequately addressed.

As I indicated in the first review, it seemed that the most important conclusion of the study is that SOM/SST cell input is more effective than PV cell input at attenuating active/supralinear excitatory responses to synaptic input in pyramidal cells. The assumption is that the differences are due to differential dendritic targeting by the two inhibitory cell types - SST inputs being stronger at more distal sites than PV inputs. However, an alternative explanation that would be more trivial could be that SST cells/axons were simply more effectively activated than PV cells/axons due to technical reasons; e.g., greater opsin expression, or stronger light stimuli, or other technical factors relating to differences in optogenetic activation. I requested that this issue be addressed more fully, either experimentally or in the Discussion. This does not seem to have been done.

In the rebuttal, the authors state: “we attempted to drive near-maximal inhibitory input from PV and SST neurons separately. This way, the effects we measure are potentially near an upper bound for each cell type.”

That sounds reasonable, but what is the evidence supporting it? Was the inhibitory output at a plateau for the two cell types (i.e., did further increases in stimulus intensity fail to increase measured inhibition?)? I could still find no explanation about how the light intensities were chosen for the two cell types. An intensity range is reported (2.21-6.25 mW/mm^2). Were the means and ranges the same for the two cell types? If the goal was to drive maximal output from both cell types, were saturating stimulus intensities delivered to both cell types? The average light intensities (and ranges) for each of the cell types should certainly be reported independently. It would also be helpful to describe the rationale for the stimulus strengths and patterns.

In the rebuttal, the authors go on to describe some of the complexities in matching inhibitory output between different presynaptic cell types, and even in defining what would constitute a match. They also state the compelling observation that SST cell inhibition was more effective despite fewer spikes in the somas of SST than PV cells. I am sympathetic to those issues/arguments and such a discussion is precisely what I was hoping the authors would add to the text when I requested it. I think such a discussion would constitute a way to address the alternative (trivial) explanation for the differential effects among cell types that I brought up earlier. I strongly encourage the authors to do so.

I’d also like to clarify one of the minor requests that I previously made. I asked: “Besides measuring x-half (offset) as an indication of threshold, could you also report the current level for the first significant non-linear jump?” What I meant to ask was whether or not those values shifted upon PV or SST cell activation? It seems like a less complicated measurement than x-half to address the issue of shifting threshold for non-linear jumps. In the rebuttal letter the authors added data for baseline current levels for the first significant jump (Reviewer Fig. 2), but did not indicate if there were optogenetically-induce shifts. Could such shift measurement be added to the ms (either in figure or text form)? Apologies for the lack of clarity previously.

Reviewer 2:

I am satisfied with the revised manuscript.

## Author Response

Synthesis of Reviews:

Synthesis Statement for Author (Required):

Both Reviewers were extremely positive about the revised manuscript. Reviewer 1 made some reasonable and pertinent requests based on their previous comments on the manuscript, so I would like you to address these before I can accept it. In particular, it would seem like the most straightforward option is to take Reviewer 1’s suggestion of adding to your Discussion section the argument you present in your rebuttal letter concerning matching inhibitory output between different cell types. Please also add the requested shift measurement (see comments below).

Reviewers’ comments

Reviewer 1:

This is a revised manuscript that I previously reviewed for the Journal of Neuroscience. I was generally enthusiastic about the previous version and the authors have made a good-faith effort to address the majority of my comments in this version. However, I do not think that the first major issue raised in my previous review has been adequately addressed.

As I indicated in the first review, it seemed that the most important conclusion of the study is that SOM/SST cell input is more effective than PV cell input at attenuating active/supralinear excitatory responses to synaptic input in pyramidal cells. The assumption is that the differences are due to differential dendritic targeting by the two inhibitory cell types - SST inputs being stronger at more distal sites than PV inputs. However, an alternative explanation that would be more trivial could be that SST cells/axons were simply more effectively activated than PV cells/axons due to technical reasons; e.g., greater opsin expression, or stronger light stimuli, or other technical factors relating to differences in optogenetic activation. I requested that this issue be addressed more fully, either experimentally or in the Discussion. This does not seem to have been done.

In the rebuttal, the authors state: “we attempted to drive near-maximal inhibitory input from PV and SST neurons separately. This way, the effects we measure are potentially near an upper bound for each cell type.”

That sounds reasonable, but what is the evidence supporting it? Was the inhibitory output at a plateau for the two cell types (i.e., did further increases in stimulus intensity fail to increase measured inhibition?)? I could still find no explanation about how the light intensities were chosen for the two cell types. An intensity range is reported (2.21-6.25 mW/mm^2). Were the means and ranges the same for the two cell types? If the goal was to drive maximal output from both cell types, were saturating stimulus intensities delivered to both cell types? The average light intensities (and ranges) for each of the cell types should certainly be reported independently. It would also be helpful to describe the rationale for the stimulus strengths and patterns.

In the rebuttal, the authors go on to describe some of the complexities in matching inhibitory output between different presynaptic cell types, and even in defining what would constitute a match. They also state the compelling observation that SST cell inhibition was more effective despite fewer spikes in the somas of SST than PV cells. I am sympathetic to those issues/arguments and such a discussion is precisely what I was hoping the authors would add to the text when I requested it. I think such a discussion would constitute a way to address the alternative (trivial) explanation for the differential effects among cell types that I brought up earlier. I strongly encourage the authors to do so.

We thank the Reviewer for their positive and constructive feedback. In our pilot studies, we determined the light intensity of 884 mW to be most effective at eliciting reliable spike responses in SST neurons, which corresponds to 6.25 mW / mm^2 for the spatial spread of light in our optical system. At lower intensities, the spike responses exhibited greater latency and fewer spikes, as one would expect. Later, when we began the PV experiments, we used the same light intensity to hold that parameter constant, and found that it evoked reliable responses in those cells as well. To more precisely describe our protocol, we have amended the Methods by adding the following text.

New text added to the Methods (page 8, line 200*):

*page and line numbers refer to the ones in the clean copy.

We used a light intensity that evoked reliable spiking responses in SST and PV cells. We used the same intensity for both SST and PV experiments (confirmed by power meter measurement to be 884 mW, which corresponds to 6.25 mW/mm2 for the spatial spread of light in our optical system).

In addition, we have added the following text to the Discussion sections to further expand our discussion on our reasoning behind the optogenetic stimulation protocol.

New text added to the Discussion (page 20, line 457):

The second limitation is that the optogenetic stimulation activated a large population of interneurons (few if any inhibitory neurons failed to respond to the light) that may not be simultaneously active under physiological conditions, and thus the condition may be considered an upper limit case. Lower levels of interneuron activation would be expected to yield smaller changes than those reported here. Relatedly, it should be noted that the level of activation of PV and SST neurons was matched in one way (IPSP amplitude as recorded somatically in pyramidal neurons, Figure 3), but it is not feasible to precisely match optogenetic control over PV and SST cells in all ways, nor is it simple to define what constitutes a “match”. We settled on the optogenetic light intensity that would reliably elicit spike responses in SST cells and used this same intensity for both SST and PV cell experiments (6.25 mW/mm2). The light pulse elicited fewer spikes in SST compared to PV cells, and the more persistent activation of PV cells was evident in the PV-IPSP shape, which exhibited a noticeably sharp offset aligned with the offset of the light stimulation. The SST-IPSP by contrast exhibited a more gradual offset that did not align with the light offset, likely due to the fewer number of evoked spikes followed by response accommodation (Figure 2). Despite these differences in their somatic spike responses to the optogenetic stimulation, the amplitudes of PV- and SST-IPSPs matched well (Figure 3B). On the one hand, this “matched” optogenetically induced IPSP size recorded at the soma may give SST cells an unfair advantage as these inhibitory inputs may be larger at the distal dendrites where SST inputs are. On the other hand, however, PV cells may not be as disadvantaged because the size of electrically induced EPSPs should be significantly reduced by the time they reach the perisomatic area where PV inputs may dominate.

I’d also like to clarify one of the minor requests that I previously made. I asked: “Besides measuring x-half (offset) as an indication of threshold, could you also report the current level for the first significant non-linear jump?” What I meant to ask was whether or not those values shifted upon PV or SST cell activation? It seems like a less complicated measurement than x-half to address the issue of shifting threshold for non-linear jumps. In the rebuttal letter the authors added data for baseline current levels for the first significant jump (Reviewer Fig. 2), but did not indicate if there were optogenetically-induce shifts. Could such shift measurement be added to the ms (either in figure or text form)? Apologies for the lack of clarity previously.

We apologize for the misunderstanding on our part. The SI values at the first significant non-linear jump for those cells that remained nonlinear did not shift upon optogenetic activation of either of the cell types. Because the electrical stimulus intensity was increased stepwise by 10 or 20 uA, it is possible that these step sizes were too coarse and failed to capture a small change in the I-O curve. Indeed, x-half was able to detect the small shift in PV but not in SST cells.

This analysis requested by the Reviewer inspired us to perform an additional analysis of x-half values in just those cells that remained nonlinear (excluding linearized cells: 6 cells with SST-activation and 2 cells with PV activation). The results remained the same for SST activation, where x-half remained unchanged by optogenetic stimulation. For PV activation, the x-half also remained unchanged, indicating that the modest difference in x-half when all cells were taken into account was driven by the ones whose responses were linearized.

We have now added the following texts to the Result section (page 15, line 347).

For those cells whose response remained nonlinear, the SI value at which the responses became supralinear did not change upon optogenetic stimulation for either cell types (SST control mean = 119.09+/-12.11, opto mean = 119.09+/-12.11, t(21) = NaN, p = 1, n = 22, paired t-test; PV control mean = 133.846+/-11.90, opto mean = 132.308+/-11.0, t(25) = 0.527, p = 0.603, n = 26, paired t-test). Thus, optogenetic activation of SST neurons, more so than PV neurons, suppressed supralinear PSPs in pyramidal neurons in layer 2/3 without affecting the gross electrical stimulus intensity threshold for supralinearity.

(page 16, line 369)

This small change in the offset was driven by the two cells whose responses were linearized by PV activation, as the offset was no longer significantly different when these cells were excluded although the trend remained the same (t(25) = -1.919, p = 0.0665, n=26). Excluding the six cells that were linearized by SST activation did not affect the x-half (t(21) = 0.1328, p = 0.89, n=22).

Reviewer 2:

I am satisfied with the revised manuscript.

We thank the Reviewer 2 for taking the time to evaluate our revised manuscript.
